# Lung inflammatory environments differentially alter mesenchymal stromal cell behavior

**DOI:** 10.1152/ajplung.00263.2019

**Published:** 2019-09-25

**Authors:** Soraia C. Abreu, Sara Rolandsson Enes, Jacob Dearborn, Meagan Goodwin, Amy Coffey, Zachary D. Borg, Claúdia C. dos Santos, Matthew J. Wargo, Fernanda F. Cruz, Roberto Loi, Michael DeSarno, Takamuru Ashikaga, Mariana A. Antunes, Patricia R. M. Rocco, Kathleen D. Liu, Jae-Woo Lee, Michael A. Matthay, David H. McKenna, Daniel J. Weiss

**Affiliations:** ^1^Department of Medicine, University of Vermont Larner College of Medicine, Burlington, Vermont; ^2^Laboratory of Pulmonary Investigation, Carlos Chagas Filho Institute of Biophysics, Federal University of Rio de Janeiro, Rio de Janeiro, Brazil; ^3^National Institute of Science and Technology for Regenerative Medicine, Rio de Janeiro, Brazil; ^4^Department of Experimental Medical Science, Lund University, Lund, Sweden; ^5^Departments of Medicine and Critical Care Medicine and the Keenan Research Center for Biomedical Science, St. Michael’s Hospital, University of Toronto, Toronto, Ontario, Canada; ^6^Department of Microbial and Molecular Genetics, Larner College of Medicine, University of Vermont, Burlington, Vermont; ^7^Department of Biomedical Sciences, University of Cagliari, Cagliari, Italy; ^8^Department of Biostatistics, University of Vermont, Burlington, Vermont; ^9^Department of Anesthesiology, Medicine and the Cardiovascular Research Institute, University of California, San Francisco, California; ^10^Department of Laboratory Medicine and Pathology, University of Minnesota, Minneapolis, Minnesota

**Keywords:** acute respiratory distress syndrome, cystic fibrosis, interleukin 6, lung injury, mesenchymal stromal (stem) cell

## Abstract

Mesenchymal stromal (stem) cells (MSCs) are increasingly demonstrated to ameliorate experimentally induced lung injuries through disease-specific anti-inflammatory actions, thus suggesting that different in vivo inflammatory environments can influence MSC actions. To determine the effects of different representative inflammatory lung conditions, human bone marrow–derived MSCs (hMSCs) were exposed to in vitro culture conditions from bronchoalveolar lavage fluid (BALF) samples obtained from patients with either the acute respiratory distress syndrome (ARDS) or with other lung diseases including acute respiratory exacerbations of cystic fibrosis (CF) (non-ARDS). hMSCs were subsequently assessed for time- and BALF concentration–dependent effects on mRNA expression of selected pro- and anti-inflammatory mediators, and for overall patterns of gene and mRNA expression. Both common and disease-specific patterns were observed in gene expression of different hMSC mediators, notably interleukin (IL)-6. Conditioned media obtained from non-ARDS BALF-exposed hMSCs was more effective in promoting an anti-inflammatory phenotype in monocytes than was conditioned media from ARDS BALF-exposed hMSCs. Neutralizing IL-6 in the conditioned media promoted generation of anti-inflammatory monocyte phenotype. This proof of concept study suggest that different lung inflammatory environments potentially can alter hMSC behaviors. Further identification of these interactions and the driving mechanisms may influence clinical use of MSCs for treating lung diseases.

## INTRODUCTION

Systemic or intratracheal administration of syngeneic, nonmajor histocompability complex (MHC) matched allogeneic, or even xenogeneic, mesenchymal stromal (stem) cells (MSCs) obtained from bone marrow, adipose tissue, and other sources, has demonstrated anti-inflammatory and disease-mitigating actions in a growing spectrum of preclinical lung disease models (reviewed in 11, 15). However, clinical investigations of MSC administration in lung diseases and critical illnesses, while demonstrating safety, have not yet shown efficacy (4, 10, 17). Notably, MSCs appear to have different mechanisms of actions in the different preclinical disease models particularly with respect to release of anti-inflammatory mediators (5, 9). Increasing evidence suggests that the MSCs respond to ambient inflammatory environments through activation of Toll-like and other damage-associated and pattern-associated cell surface receptors expressed by the MSCs by releasing patterned sets of anti-inflammatory mediators, including anti-inflammatory cytokines, antibacterial peptides, and miRNA-containing microsomal particles (16). However, the effects of different inflammatory environments on MSC behavior have been predominantly characterized by in vitro model systems, and there is less information as to what effects specific in vivo lung inflammatory environments have on MSC actions (3). We hypothesized that different in vivo environments significantly alter MSC responses and thus potential therapeutic efficacy. One recent study demonstrated effects of different lung injury models in mice on the lung proteome and correlative effects on potential protective actions of MSC administration (7). Thus, understanding in vivo inflammatory effects on MSCs, particularly with respect to the release of anti-inflammatory mediators, could provide important mechanistic information as well as a method to optimally precondition the MSCs to maximize the potency of the cells, conditioned media, or cell-derived extracellular vesicles for a given disease indication.

Therefore, in this proof of concept study we used clinical bronchoalveolar lavage fluid (BALF) samples as a surrogate of the in vivo inflammatory lung environment from several different inflammatory lung conditions, including the acute respiratory distress syndrome (ARDS), to assess for effects on hMSC gene and mRNA expression and for effects of BALF-exposed hMSC-conditioned media on monocyte behavior. Individual rather than pooled samples were utilized to determine both intra- and interdisease effects.

## METHODS

### 

#### Cells.

MSCs derived from multiple adult bone marrow of healthy volunteers (hMSCs) were obtained from the National Heart, Lung, and Blood Institute’s (NHLBI) Production Assistance for Cellular Therapies program (University of Minnesota) and cultured in DMEM-EBSS medium with 2 mM l-glutamine, 100 U/mL penicillin, 100 μg/mL streptomycin, and 20% fetal bovine serum (Gibco, Invitrogen, Carlsbad, CA) (14). Cells were used at passage six or lower and maintained in culture at confluency no greater than 70%. Purity was determined by expression of Sca-1, CD106, CD29, and absence of CD11b, CD11c, CD34, CD45, and the ability to differentiate into chondrocytes and adipocytes in vitro. Mouse RAW 264.7 monocytes (ATCC) were cultured in DMEM medium with 2 mM l-glutamine, 100 U/mL penicillin, 100 μg/mL streptomycin, and 10% fetal bovine serum.

#### Human bronchoalveolar lavage fluid.

Human BALF was obtained prospectively under an existing Institutional Review Board (IRB) protocol (CHRMS 03-191) from patients with cystic fibrosis (CF) or other lung diseases undergoing diagnostic bronchoscopies during acute respiratory exacerbations (denoted as non-ARDS BALF samples) at the University of Vermont Medical Center (UVMMC, Burlington, VT). ARDS BALF was obtained from a repository of samples collected as part of unrelated clinical investigations conducted by the National Heart, Lung, and Blood Institute (NHLBI), specifically from the APC trial for ARDS. IRB approvals were in place for collection of these samples at University of California San Francisco (UCSF). All BALF samples were de-identified and numerically coded. All human participants provided written informed consent. Primary BALF samples collected at UVM were centrifuged at 500 *g* for 5 min to remove any remaining viable cells or cell debris. ARDS samples obtained from UCSF were from mini-BAL and generally obtained 48 h after enrollment in clinical trials. A total of nine separate ARDS and six non-ARDS clinical samples were evaluated for the different studies. Delineation of each sample and how it was utilized is shown in Table 1 as is the etiology of ARDS for the ARDS samples.

**Table 1.** Delineation of each sample and how it was utilized in this study

	Location Collected	Primary ARDS Etiology	Study
ARDS BALF sample			
A1	USC	Aspiration	Dose-dependency RT-PCR
A2	UCSF	Aspiration	Dose-dependency RT-PCR
A3	USC	Aspiration	Dose-dependency RT-PCR
A4	Fresno, CA	Aspiration	Dose-dependency RT-PCR, microarray
A5	USC	Pneumonia	Dose-dependency RT-PCR, microarray, 24 h RT-PCR
A6	USC	Pneumonia	Early time RT-PCR, microarray, 24 h RT-PCR
A7	Oregon	Other	Early time RT-PCR, microarray, monocyte phenotype
A8	UCSF	Pneumonia	Monocyte phenotype, 5 and 24 h RT-PCR
A9	Oregon	Other	Monocyte phenotype, 5 and 24 h RT-PCR
Non-ARDS BALF sample			
1	UVMMC	CF	Dose-dependency RT-PCR
2	UVMMC	CF	Dose-dependency RT-PCR, microarray
3	UVMMC	COPD	Dose-dependency RT-PCR, microarray
4	UVMMC	CF	Early and late time RT-PCR, microarray
5	UVMMC	Sarcoid	Early time RT-PCR, microarray
6	UVMMC	Non-CF bronchiectasis	Monocyte phenotype

ARDS, acute respiratory distress syndrome; BALF, bronchoalveolar lavage fluid; CF, cystic fibrosis; COPD, chronic obstructive pulmonary disease; USC, Univ. of Southern California; UCSF, Univ. of California, San Francisco; UVMMC, University of Vermont Medical Center.

#### In vitro incubation of hMSCs with BALF samples.

hMSCs were plated in 24-well plates at a concentration of 25,000 cells/mL (~50% confluence) in hMSC basal medium (DMEM-EBSS medium with 2 mM l-glutamine, 100 U/ml penicillin, 100 μg/mL streptomycin, and 20% fetal bovine serum (Gibco, Invitrogen, Carlsbad, CA). After overnight adherence, cells were washed once with PBS and incubated for 4 h in either basal medium, serum-free medium, or serum-free medium containing 10%, 20%, 50%, or 100% vol:vol concentration of the BALF. In a parallel series of studies, the concentration BALF in serum-free medium was held constant at 25%, and hMSCs were incubated for either 1, 3, 5, or 24 h. After incubations, supernatants were removed by aspiration, and cells were washed twice with PBS. Fresh serum-free media was placed and cells incubated for 24–48 h to collect conditioned media.

#### RNA isolation, cDNA synthesis, and RT-qPCR.

In experiments for RT-PCR and microarray analyses, following respective incubations with BALF samples and subsequent washing, cells were incubated with TriReagent (Molecular Research Center, Inc.). RNA was extracted following the manufacturer’s instructions, cleaned using PrepEase columns (USB), and eluted in 60 µL nuclease-free water. Total RNA purity and concentration were assessed by spectrophotometry via Nanodrop (ThermoFisher). cDNA was synthesized using iScript cDNA synthesis kit (Biorad) according to manufacturer’s protocol. All RT-qPCR was performed using IQ-SYBR Green Supermix (BioRad) according to manufacturer’s instructions on a CFX96 Real-Time System (BioRad). Primers are listed in Supplemental Table S1 **(**https://doi.org/10.6084/m9.figshare.9744791**)**.

#### Microarray analyses.

Samples were run on the Illumina HumanHT-12_v4 bead array at the University of Minnesota Microarray Core Facility. The data were compressed using Illumina’s GenomeStudio version 1.9.0. and imported into Partek for multi- and univariate analyses. Raw GeneChip data (one DAT file for each chip) included a collection of images, one for each probe and chip. Each image was summarized by Affymetrix GCOS software by using one probe intensity (in CEL files, one per chip). No background correction was performed. Information from multiple probes was combined to obtain a single measure of expression for each probe set and sample. Probe level intensities were calculated using the Robust Multichip Average (RMA) algorithm, including background correction, normalization (quantile), and summarization (median polish), for each probe set and sample, as is implemented in Partek Genomic Suites, version 6.6 (Copyright 2009, Partek Inc., St. Louis, MO). Sample quality was assessed based on the 3′:5′ ratio (3′ arrays only), relative log expression (RLE), and normalized unscaled standard error (NUSE).

#### Assessment of hMSC-conditioned media effects on pro-inflammatory versus anti-inflammatory monocyte phenotype polarization.

hMSCs were incubated with 50% BALF for 3 h, washed with PBS, and then incubated with DMEM/EBSS medium without fetal bovine serum for 24 h. The conditioned media was collected and mouse RAW 264.7 monocytes cultured either with regular medium or the different-conditioned media in a standard 12-well plate for 24 and 48 h. The supernatants were then collected at each time point after co-incubation and assayed for levels of interleukin 10 (IL-10) and tumor necrosis factor-α (TNF-α) by ELISA (R&D Systems). Levels of IL-6 released into the conditioned media were measured by ELISA (R&D Systems), and in a parallel series of incubations, neutralizing IL-6 antibody (5 μg/mL, Biolegend) was added to the conditioned media before incubation with the cells.

#### Statistical analyses.

Differences between mRNA expression were assessed by Kruskal-Wallis test with Dunn’s multiple comparison test. Differences in protein levels of IL-6, IL-10, and TNF-α were assessed by one-way ANOVA with Fisher’s LSD test or Dunn’s post hoc analyses. For microarray analyses, principal component analysis (PCA) was used to look for outlier samples that would potentially introduce latent variation into the analysis of differential expression across sample groups. Univariate linear modeling of sample groups was performed using ANOVA as implemented in Partek Genomic Suites. The magnitude of the response (fold change calculated using the least square mean) and the *P* value associated with each probe set and binary comparison was calculated, as well a “step-up,” adjusted *P* value for the purpose of controlling the false discovery rate (2). ANOVA was also performed to detect alternative splicing. Functional enrichment was performed using ingenuity pathways analysis (8) and through the NIH David software program to identify biologic processes associated with differentially expressed genes (6). In addition, a subset of the microarray expression data set, with 25% dilution BALF treatment only, was used for follow-up statistical analyses. These analyses were done to test for statistically significant differences between BALF groups and between time points (*t* = 1, 3, 5 h) across groups, and to test for statistically significant group by time point interactions, to determine whether the effect of time differed significantly between groups. Additional analyses were done within each group, which also incorporated the baseline time point (*t* = 0) to further evaluate the effect of time. These analyses were done using mixed model repeated measures analyses of variance. The sets of raw *P* value results for all genes from these analyses were corrected for false discovery rate (FDR) by using the Hochberg-Benjamini method. FDR-corrected *P* value less than significance level alpha = 0.05 were considered statistically significant. These analyses were conducted using SAS statistical software, version 9.2, SAS Institute, Inc., Cary, NC.

## RESULTS

**Fig. 1.**
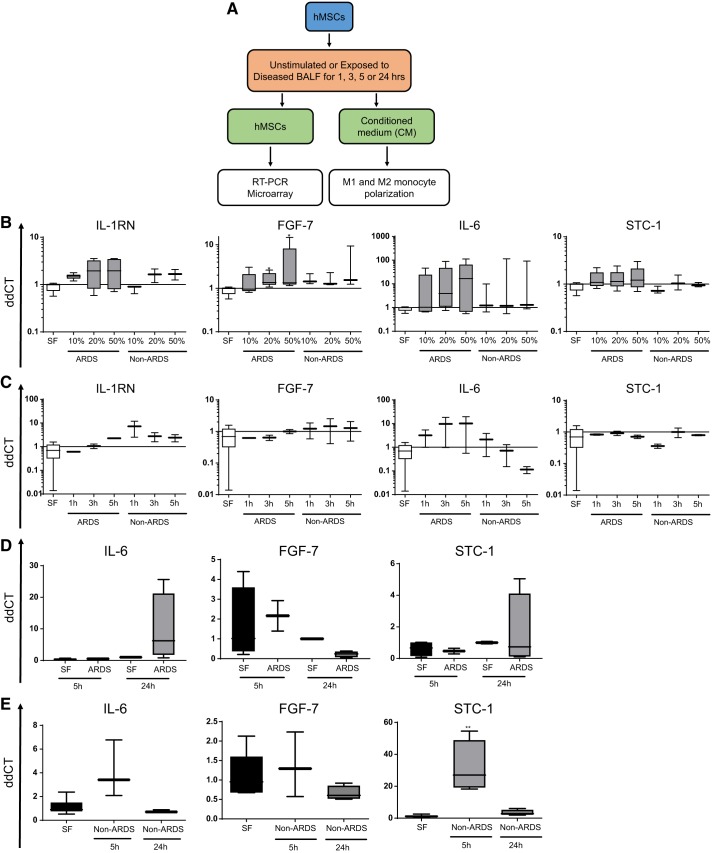
*A*: schematic of studies. *B* and *C*: short-term incubation of human mesenchymal stromal cells (hMSCs) with bronchoalveolar lavage fluid (BALF) from acute respiratory distress syndrome (ARDS) or other lung diseases (non-ARDS) alters mRNA levels of several secreted mediators in a dose- and time-dependent manner (10%, 20%, or 50% vol:vol for 4 h and 25% vol:vol for 1, 3, or 5 h, respectively). Data are presented as box and whisker plots with minimum to maximum of relative gene expression compared with basal levels in hMSCs cultured under standard conditions (set to 1); *n* = 7 serum-free control, 5–6 ARDS, and 2–3 non-ARDS samples for the dose studies, and *n* = 7 serum-free control, 2 ARDS, and 2 non-ARDS samples for the kinetic studies. *D* and *E*: longer-term incubation of hMSC with BALF from ARDS or non-ARDS alters mRNA levels of genes encoding for several secreted mediators in a dose- and time-dependent manner. Data are presented as box and whisker plots with minimum to maximum of relative gene expression compared with basal levels in hMSCs cultured under standard conditions (set to 1). SF indicates hMSCs incubated with serum-free medium alone; *n* = 2 ARDS (4 technical replicates), 3–4 non-ARDS samples (1 technical replicate), and 1–6 serum-free controls (1–4 replicates) for 5-h incubations, and *n* = 4 ARDS (2 technical replicates), 3–4 non-ARDS (1 technical replicate), and 1–6 serum-free controls (1–2 technical replicate) for 24-h incubations. **P < 0.01 compared with control.

The experimental schematic is depicted in Fig. 1*A*. To initially determine differential responses of hMSCs to the different disease BALF samples, the expression of genes encoding for known MSC-secreted mediators [angiopoietin 1 (Ang-1), fibroblast growth factor 7 (FGF-7), interleukin 1 receptor antagonist (IL-1RN), IL-6, stanniocalcin-1 (STC1), transforming growth factor-β (TGFβ), and tumor necrosis factor-inducible gene 6 protein (TSG-6)] in response to *1*) increasing “doses” of BALF and *2*) over time was assessed. Notably, longer (4-h) incubation with 100% BALF, particularly from CF patients, was often toxic to the hMSCs. This was not attributable to presence of bacteria such as *P. Aeruginosa* or *S. Aureus*. However, dilutions at 50%, 20%, and 10% had no discernible effects on the cells (Fig. 1*B*). There was a dose-dependent trend towards increase in mRNA for IL-1RN (*n* = 6), FGF-7 (*n* = 5), and IL-6 (*n* = 5) in hMSCs incubated with BALF obtained from five to six separate ARDS patients. The increase in IL-6 mRNA was particularly notable. Also noteworthy was the general similarity in increase across BALF from different ARDS clinical samples, suggesting a common mediator or mediators. While STC-1 (*n* = 5) mRNA expression was detected, minimal change compared with baseline was observed in the following exposures. Similar patterns, including trends towards dose-dependent increases in IL-6 and also IL-1RN and FGF-7 were observed in mRNA levels from hMSCs exposed to two separate CF and one chronic obstructive pulmonary disease (COPD) BALF samples. As with exposure to the ARDS samples, there was an overall consistency between results obtained from the separate BALF samples. Further, as with exposure to the ARDS BALF samples, minimal expression of Ang-1, TGFβ, or TSG-6 mRNA was observed under any condition (data not shown).

To assess early changes in mRNA expression over time, hMSCs were incubated for 1, 3, or 5 h with 25% dilution BALF of two additional BALF samples, each obtained from ARDS or non-ARDS (1 CF, 1 sarcoid) patients (Fig. 1*C*). In contrast to relatively similar patterns observed with differing dose, distinct patterns over time were observed between hMSCs exposed to ARDS versus non-ARDS BALF samples. A time-dependent increase in IL-6 mRNA was observed in ARDS-exposed hMSCs versus a time-dependent decrease observed following hMSC exposure to the other BALF samples. In contrast, more robust time-dependent IL-1RN mRNA expression was observed following exposure to the non-ARDS BALF samples as compared with ARDS BALF. No significant early time effects were observed in STC-1 or FGF-7 mRNA expression (Fig. 1*C*) or in Ang-1, TGFβ, or TSG-6 mRNA expression (not shown). Again noted was a disease-specific consistency in effects across the different clinical BALF samples.

To assess longer term effects on mRNA expression, hMSCs were exposed to ARDS BALF samples for either 5 h (*n* = 2) or 24 h (*n* = 4) (Fig. 1*D*). In this series of studies, less robust increase in mRNA levels for IL-6 and IL-1RN was observed at 5 h, but in each case increased IL-6 mRNA was observed at 24 h. Levels of FGF-7 mRNA trended towards lower expression at 24 versus 5 h, whereas STC-1 mRNA levels trended towards increase at 24 versus 5 h. These changes were somewhat consistent between the four individual clinical specimens. In contrast, mRNA levels of Ang-1, TGFβ, and TSG-6 remained generally at baseline and showed more variability between clinical samples. After similar hMSC exposure to an additional other BALF sample for 5 and 24 h, levels of IL-6 mRNA were significantly increased following 5 h but returned towards baseline at 24 h (Fig. 1*E*). In contrast to the first round of studies following exposure to CF and sarcoid BALF samples (Fig. 1*C*), there was a significant increase in STC-1 mRNA at 5 h that also returned towards baseline at 24 h. No significant changes were observed following either 5 or 24 h exposure in mRNA levels of Ang-1 or FGF-7, which remained at baseline. An increase in TGFβ and TSG-1 mRNA was observed at 5 h and maintained at 24 h for TSG-1.

To further assess BALF effects on gene expression following different dose and time exposures, microarray analyses were performed following exposure to three of the ARDS and four of the non-ARDS BALF samples used for the above qPCR studies. Principal component analyses demonstrated significant differences between time points (*n* = 2 each for ARDS and non-ARDS BALF samples) but less significant differences between concentrations (*n* = 2 each for ARDS and other BALF samples) (data not shown). As such, heat maps and subsequent analyses were only generated from the time point data. Follow-up analyses of microarray expression were done using a subset of the data for the 25% dilution BALF treatment only. For the analyses done across both groups, the only significant results found were for main effect of time, between all three time points included in these analyses (*t* = 1, 3, 5 h). For these analyses, there were 29 genes with FDR-corrected *P* values < 0.05 for main effect of time. For the analyses done with each group, again, the only significant results found were for effect of time, between all four time points included in these analyses (*t* = 0, 1, 3, 5 h). For the analysis within ARDS group, there were 13 genes with FDR-corrected *P* values < 0.05 for effect of time, while for the analysis within other BALF group, there were 33 genes with such *P* values results.

**Fig. 2.**
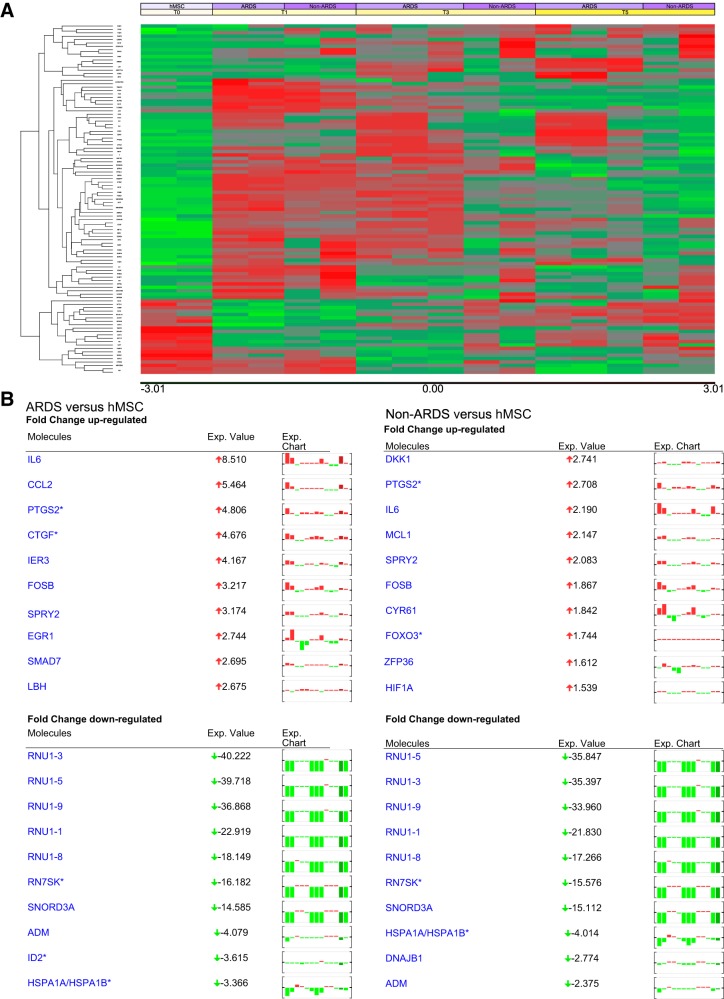
*A*: representative heat maps of time-dependent human mesenchymal stromal cell (hMSC) gene expression following exposure to 2–3 acute respiratory distress syndrome (ARDS) and 3 non-ARDS bronchoalveolar lavage fluid (BALF) samples. *B*: depiction of 10 genes with highest time-dependent up- or downregulation.

**Fig. 3.**
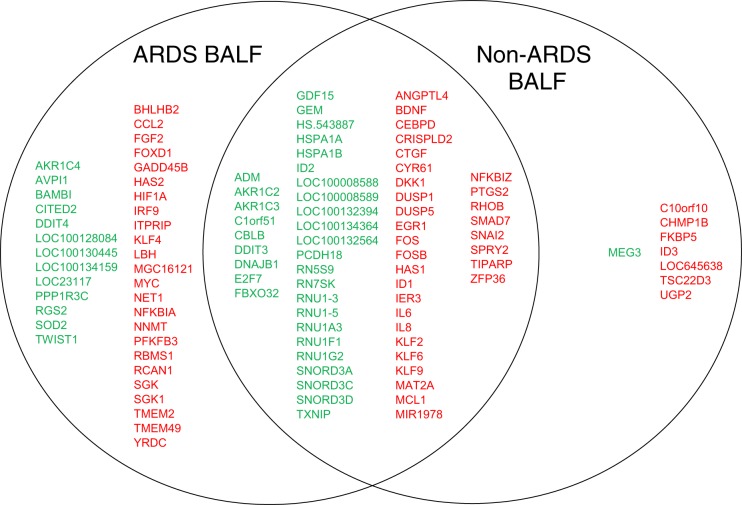
Venn diagram showing overlap in gene expression. Values depicted are from one representative sample each of non-acute respiratory distress syndrome (ARDS) vs. ARDS bronchoalveolar lavage fluid (BALF)-exposed human mesenchymal stromal cells (hMSCs).

A representative heat map depicting gene expression following either 1, 3, or 5 h hMSC exposure to a representative ARDS or non-ARDS (CF) BALF sample is shown in Fig. 2*A* and demonstrates a number of interesting observations. Initially, there is a widespread early (1 h) up- or downregulation in gene expression in both ARDS and non-ARDS BALF-exposed hMSCs compared with nonexposed control hMSCs. While there are some differences between the clinical samples, the overall patterns are similar within each disease category. Moreover, the overall early patterns are generally similar between ARDS versus non-ARDS BALF-exposed hMSCs. However, over longer time periods of exposure, particularly after 5 h, there appear both individual as well as disease-specific changes in gene expression. Assessing the 10 genes with the highest fold up or downregulation over the 5-h exposure period demonstrated similar patterns in downregulated gene expression between individual patient samples and between disease categories, particularly among members of the RNU family of small nuclear RNA (snRNA) component of the spliceosome (involved in pre-mRNA splicing) (Fig. 2*B*). In contrast, nearly completely different disease-specific patterns of the 10 highest upregulated genes were observed. The only common genes upregulated following exposure to both ARDS and non-ARDS BALF samples were IL-6, prostaglandin-endoperoxide synthase 2 (PTGS2), and the sprout homolog SPRY2. A broader range of up- and downregulated genes (≥2-fold change) is depicted in Fig. 3. There is both overlap and disease-specific patterns observed. No significant up or downregulation was observed in gene expression of Ang-1, FGF-7, IL-1RN, STC-1, TGFβ, or TSG-6 following exposure to the different BALF samples at any of the early time points assessed.

**Fig. 4.**
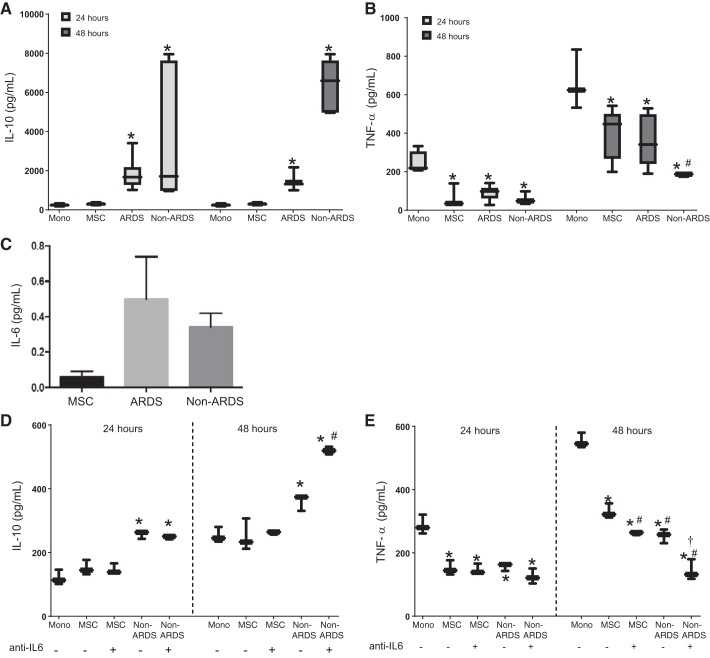
Exposure to bronchoalveolar lavage fluid (BALF) from acute respiratory distress syndrome (ARDS) (*n* = 2) or non-ARDS (*n* = 2) patients promotes increased IL-10 (*A*), decreased TNF-α secretion (*B*), and a trend towards increase in IL-6 released into conditioned media (*C*). *D*: neutralization of IL-6 increase IL-10 secretion. *E*: neutralization of IL-6 decrease TNF-α secretion. Data are presented as box and whisker plots with minimum to maximum of 12 experimental replicates for ARDS and 6 experimental replicates for non-ARDS samples. *P < 0.05 compared with control. #*P* < 0.05 compared with ARDS. †*P* < 0.05 compared with non-ARDS.

To assess a functional effect following exposure to the different BALF samples, hMSCs were incubated overnight with BALF from two ARDS and two non-ARDS (1 CF, 1 non-CF bronchiectasis) samples, washed, and then incubated overnight again in serum-free media. Effects on monocyte pro-inflammatory versus anti-inflammatory phenotype were then assessed by incubating cultured monocytes overnight with the different-conditioned media samples (Fig. 4, *A* and *B*). Notably, anti-inflammatory phenotype, observed by the higher levels of IL-10, was robustly induced in the monocytes, more so following exposure to conditioned media obtained from hMSCs previously exposed to the non-ARDS fluid samples. As IL-6 gene and mRNA levels were consistently induced by exposure to both ARDS and non-ARDS BALF samples, levels of IL-6 were assessed in conditioned media by ELISA, and a trend towards increase was observed following overnight exposure to both ARDS and non-ARDS BALF samples (Fig. 4*C*). To clarify whether IL-6 played a role in induction of M2 phenotype, monocytes were incubated overnight with conditioned media obtained from BALF-exposed hMSCs in the presence of a neutralizing anti-IL6 antibody. Surprisingly, this resulted in more robust induction of M2 phenotype (Fig. 4, *D* and *E*).

## DISCUSSION

This proof of concept study demonstrates that exposure of human bone marrow–derived MSCs, in this case clinical-grade MSCs obtained from the NHLBI PACT program, with different clinical BALF samples obtained from a heterogeneous mix of patients with either ARDS, respiratory exacerbations of CF, or other respiratory diseases, resulted in a range of changes in the hMSCs. Gene expression and mRNA levels changed in a dose- and time-dependent manner following exposure. Acknowledging the small number of samples from some of the different disease categories, there were some suggestive similarities and differences between the disease categories. Interestingly, despite the clinical heterogeneity of the individual samples, there were some similar patterns of gene and mRNA expression between different patient samples within a disease category, notably ARDS and non-ARDS BALF exposures. Of the different potential mediators affected by BALF exposures, IL-6 gene and mRNA levels were most prominently increased by both ARDS and other BALF exposures. The specific BALF mediators responsible for this are at present unknown, but previous work had demonstrated that IL-1β in ARDS BALF induced IL-6 expression in fibroblasts (13). Interestingly, neutralizing IL-6 in hMSC-conditioned media following exposure of hMSCs to non-ARDS BALF augmented promotion of anti-inflammatory phenotype. These are all intriguing proof of concept initial findings and support the central hypothesis that in vivo lung inflammatory environments can differentially affect a range of MSC behaviors. These findings also coincide with a currently small but growing literature demonstrating differential effects of either BALF or serum obtained from models of preclinical lung diseases or from ARDS patients on MSC behaviors (1, 3, 7, 12). Notably, hMSCs exposed to pooled ARDS BALF samples suppressed cytokine production, increased M2 monocyte marker expression, and augmented phagocytic capacity of human monocytes, effects were partially mediated by CD44-expressing EVs produced by the exposed MSCs (12). Other recent data demonstrated different proteomic profiles in hMSCs exposed to human ARDS BALF and correlative effects on potential protective actions of hMSC administration in a mouse model of acute lung injury (7). Recent studies have also demonstrated that MSCs previously exposed to either serum or BALF obtained from mice with experimentally induced allergic airways inflammation have differential protective effects when administered back to mice with the same type of allergic airways inflammation (1). Importantly, all of these studies validate the use of BALF as well as serum as effective clinical surrogates for the inflammatory lung environment.

One of the strengths of these observations is the analysis of individual BALF samples. This allows a broad understanding of disease-specific effects, despite clinical heterogeneity within any disease category. However, this also resulted in limitations to this initial study. Relatively small amounts of BALF samples were obtained from each study patient, and there are also only limited numbers of representative patients in certain disease categories. It was thus not always possible to perform all the desired assays on each clinical BALF sample; however, as delineated in Table 1, overlapping assays were performed when feasible. There are also no healthy control (normal volunteer) BALF samples included. We are currently amassing a larger BALF sample pool, and ongoing studies will allow further analyses of larger samples pools, including the ability to assess age and gender effects. Future studies will also assess effects of BALF-exposed hMSC-conditioned media and extracellular vesicles on behaviors of primary rather than monocyte cell lines and also on other immune cell populations. Another critical point when considering MSC as a potential cell-based therapy for lung diseases is the donor phenotype. In this study, we were using multiple donors in order to have a biological representability. Comparison of BALF effects on MSCs obtained from different donors needs to be further studied.

The data presented in this study suggest that the inflammatory microenvironment present in lung disease patients, including ARDS and CF, alters MSC behaviors. Notably, other changes in the microenvironment such as different oxygen partial pressures (18), substrate stiffnesses (19), and mechanical stretch (20) may also affect MSC behaviors and are worthy of further investigation. Further understanding the effects of in vivo environments on MSC behaviors will provide critical information that will help guide understanding MSC potency and guide preconditioning of MSCs for any potential therapeutic application. This information may also better define patient populations more likely to respond to MSC-based cell therapies.

## GRANTS

D. J. Weiss is supported by Cystic Fibrosis Foundation Research Grants WEISS15XX0 and WEISS16GO. S. Rolandsson Enes is supported by a Marie Curie Post-doctoral Research Fellowship (RESPIRE3) from the European Respiratory Society and the European Union’s H2020 Research and Innovation Programme (Marie Sklodowska-Curie Grant Agreement No. 713406). M. A. Matthay is supported by National Heart, Lung, and Blood Institute (NHLBI) Grants U01 HL123004 and R42 HL126456. D. H. McKenna is supported by the NHLBI PACT program, University of Minnesota, Molecular and Cellular Therapeutics, contract HHSN268201000008'.

## DISCLOSURES

No conflicts of interest, financial or otherwise, are declared by the authors.

## AUTHOR CONTRIBUTIONS

M.G., Z.D.B., F.F.C., R.L., M.A.M., D.H.M., and D.J.W. conceived and designed research; S.C.A., S.R.E., J.D., M.G., A.C., Z.D.B., F.F.C., R.L., M.A.A., M.A.M., D.H.M., and D.J.W. performed experiments; S.C.A., S.R.E., J.D., M.G., A.C., Z.D.B., C.C.d.S., M.J.W., F.F.C., R.L., M.D., T.A., M.A.M., D.H.M., and D.J.W. analyzed data; S.C.A., J.D., M.G., A.C., Z.D.B., F.F.C., R.L., M.A.A., P.R.M.R., K.D.L., J.-W.L., M.A.M., D.H.M., and D.J.W. interpreted results of experiments; S.R.E., M.A.M., D.H.M., and D.J.W. prepared figures; S.R.E., D.H.M., and D.J.W. drafted manuscript; S.C.A., S.R.E., J.D., M.G., A.C., Z.D.B., C.C.d.S., M.J.W., F.F.C., R.L., M.A.A., P.R.M.R., K.D.L., J.-W.L., M.A.M., D.H.M., and D.J.W. edited and revised manuscript; S.C.A., S.R.E., J.D., M.G., A.C., Z.D.B., C.C.d.S., M.J.W., F.F.C., R.L., M.A.A., P.R.M.R., K.D.L., J.-W.L., M.A.M., D.H.M., and D.J.W. approved final version of manuscript.
